# Downstream Behavioral and Electrophysiological Consequences of Word Prediction on Recognition Memory

**DOI:** 10.3389/fnhum.2019.00291

**Published:** 2019-08-28

**Authors:** Ryan J. Hubbard, Joost Rommers, Cassandra L. Jacobs, Kara D. Federmeier

**Affiliations:** ^1^Department of Psychology, University of Illinois, Urbana-Champaign, IL, United States; ^2^Beckman Institute for Advanced Science and Technology, University of Illinois, Urbana-Champaign, IL, United States; ^3^Centre for Cognitive Neuroimaging, Donders Institute for Brain, Cognition and Behaviour, Radboud University Nijmegen, Nijmegen, Netherlands; ^4^Department of Psychology, Center for Mind and Brain, University of California, Davis, Davis, CA, United States; ^5^Program in Neuroscience, University of Illinois, Urbana-Champaign, IL, United States

**Keywords:** language comprehension, prediction, false memory, recognition, ERP

## Abstract

When people process language, they can use context to predict upcoming information, influencing processing and comprehension as seen in both behavioral and neural measures. Although numerous studies have shown immediate facilitative effects of confirmed predictions, the downstream consequences of prediction have been less explored. In the current study, we examined those consequences by probing participants’ recognition memory for words after they read sets of sentences. Participants read strongly and weakly constraining sentences with expected or unexpected endings (“I added my name to the list/basket”), and later were tested on their memory for the sentence endings while EEG was recorded. Critically, the memory test contained words that were predictable (“list”) but were never read (participants saw “basket”). Behaviorally, participants showed successful discrimination between old and new items, but false alarmed to the expected-item lures more often than to new items, showing that predicted words or concepts can linger, even when predictions are disconfirmed. Although false alarm rates did not differ by constraint, event-related potentials (ERPs) differed between false alarms to strongly and weakly predictable words. Additionally, previously unexpected (compared to previously expected) endings that appeared on the memory test elicited larger N1 and LPC amplitudes, suggesting greater attention and episodic recollection. In contrast, highly predictable sentence endings that had been read elicited reduced LPC amplitudes during the memory test. Thus, prediction can facilitate processing in the moment, but can also lead to false memory and reduced recollection for predictable information.

## Introduction

The process of prediction has been suggested to play a role in many areas of cognition and behavior, with some arguing that one of the core functions of the brain is to use previously learned associations and top-down control to predict future events (Bar, 2007, 2009; Bubic et al., 2010; Clark, 2013). This function of predicting upcoming information may play a particularly important role in language comprehension (Federmeier, 2007; Kuperberg and Jaeger, 2016), as incoming linguistic information must be processed rapidly. Essentially, by using the bottom-up sensory information provided by written and spoken words, combined with previously learned world knowledge, semantic, and syntactic information, the brain can quickly create and continuously update a representation of likely upcoming linguistic information, which facilitates processing when this information is encountered.

As evidence of the impact of predictability on language comprehension, behavioral work has shown that words that are highly predictable and fit into the ongoing sentence context are processed more rapidly than less predictable words (West and Stanovich, 1978; Fischler and Bloom, 1979; Schuberth et al., 1981; Schwanenflugel and LaCount, 1988; Duffy et al., 1989; Simpson et al., 1989; Hess et al., 1995). Similarly, eye-tracking studies have demonstrated that predictable words are anticipated and read more quickly than unpredictable words (Ehrlich and Rayner, 1981; Altmann and Kamide, 1999; Frisson et al., 2005; Kamide, 2008). Research using event-related potentials (ERPs) has identified that the predictability of words affects the amplitude of the N400, a centroparietal negativity peaking around 400 ms that is thought to index access of semantic memory (Kutas and Hillyard, 1984; Federmeier and Kutas, 1999; Wlotko and Federmeier, 2007, 2012; Kutas and Federmeier, 2011; DeLong et al., 2014). Additionally, unexpected but plausible words that disconfirm a prediction elicit a late, frontally-distributed positivity, which has been hypothesized to index a revision process of some kind (Federmeier et al., 2007; Otten and Van Berkum, 2008; DeLong et al., 2011, 2014; Thornhill and Van Petten, 2012).

There is thus substantial evidence that predictability can lead to facilitated processing of expected information when it is encountered. There are also consequences of processing inputs that violate predictions, as indexed by the late frontal positivity. Do these consequences that are evident in ERPs have corresponding behavioral costs? In early work using lexical decision tasks, identification of predictable words was consistently faster than unpredictable words, but prediction violations did not always lead to response slowing when compared to “baseline” conditions, which varied across the literature (Schuberth and Eimas, 1977; Fischler and Bloom, 1979; Schwanenflugel and Shoben, 1985). Other recent work, in which subjects read sentences at their own pace while eye movements were tracked, reported no evidence of slowing or an increase in re-reading for unexpected words (Luke and Christianson, 2016; Frisson et al., 2017). Therefore, across multiple behavioral paradigms of language processing, convincing evidence of behavioral costs associated with prediction violations has been lacking.

In addition, behavioral and electrophysiological effects of prediction have predominantly been measured at the time of encountering the predicted or unexpected stimulus. Although this has been useful for identifying the immediate effects of prediction, it leaves open what downstream effects confirmed or disconfirmed predictions might have on later cognition. In order to investigate these potential downstream effects, the present study tested participants’ episodic memory for sentence final words of sentences that varied in contextual constraint. The memory test contained words that had been highly predictable, weakly predictable, or unexpected. This allowed for a comparison of the downstream effects of having predictions confirmed or disconfirmed. Critically, the test also included words that were likely to have been predicted but were never actually observed during reading (because the sentence instead had ended with an unexpected word); we will refer to these items as lures.

In addition to behavioral memory measures, the present study recorded EEG to further probe how predictability influences memory processes. Examining ERPs during the memory test allowed us to draw inferences about the neurocognitive processes involved in successfully recognizing, or false alarming to, predictable and unexpected words. Previous studies have identified two major components associated with recognition memory (Rugg and Curran, 2007)—the N400, which has been linked to conceptual fluency or familiarity (Paller and Kutas, 1992; Curran, 2000, 2004; Voss and Federmeier, 2011), with greater familiarity leading to smaller N400s, and the LPC, a left-lateralized posterior component temporally extending from 500 to 800 ms, which is related to recollection or retrieval of more detailed episodic information (Düzel et al., 1997; Rugg et al., 1998; Woodruff et al., 2006: Yu and Rugg, 2010), with greater recollection eliciting more positive LPCs. The amplitudes of these ERP components during the memory test may differ based on the prior predictability of the words or the constraint of the sentences they were presented in, which would provide information about the state of the representations of these items.

Two main issues were of interest: first, we compared memory for predictable words and unexpected words. Here, context-driven prediction could influence the encoding of information into long-term memory by modulating the level of attention given to the predictable or unpredictable information that is being encoded (Craik et al., 1996). Paying more attention to certain stimuli could modulate the depth or level of processing, leading to a more stable and persistent memory representation (Craik and Lockhart, 1972; Craik and Tulving, 1975). In eye-tracking experiments with natural reading, individuals spend less time looking at and exhibit fewer regressions to predictable words (Ehrlich and Rayner, 1981), suggesting they may, in fact, pay less attention to them. Rommers and Federmeier (2018a), investigating ERP repetition effects for previously predictable and unpredictable words, also found that previously predictable words showed reduced downstream repetition effects, suggesting that prediction can lead to an impoverished initial representation. In the case of unpredictable words, some evidence points toward attentional enhancement of encoding: an item in a list of words that is physically or semantically distinct from the others will be more likely to be recalled (Von Restorff, 1933), unexpected sentence endings draw more attention away from and lead to disruption of serial recall (Röer et al., 2019), and unexpected or error-related events modulate early attention-related ERPs (Wills et al., 2007), suggesting that distinctive, unpredictable events might be more attended to and then more easily remembered. Indeed, studies have reported better recognition memory performance for sentence endings that had been unpredictable (compared with predictable endings), supporting the idea that such words are encoded more strongly (Corley et al., 2007; Federmeier et al., 2007). We further probed the memory processes underlying the recognition of previously predictable and previously unexpected words. In particular, if previously encountered sentence endings increase conceptual priming at test, they should show a reduced N400, whereas if they increase recollection processes, they should elicit an enhanced LPC.

We were also interested in the responses to the lures. If prediction during sentence comprehension leads to pre-activation of information associated with an upcoming word, then participants may show greater false alarms to lures as compared to completely new items. This would constitute a cost of prediction, in that lingering representations can cause false recognition. Alternatively, if the prediction disconfirmation leads to strong revision processes that suppress previously expected information, participants may show fewer false alarms to lures as compared to completely new items. Previous studies have employed an implicit memory paradigm in which participants predict a high cloze ending, are given an unexpected ending, and then must complete a mid-cloze sentence that could potentially be completed by a previous high-cloze or unexpected ending (Hartman and Hasher, 1991; Lorsbach et al., 1996; Hasher et al., 1997). These studies have focused mainly on inhibition and control processes; however, they have demonstrated that individuals tend to retain the expected but disconfirmed endings in some form. In terms of explicit memory, classic studies using the Deese-Roediger-McDermott (DRM) paradigm have shown that individuals will recall an unstudied semantic associate (e.g., “sleep”) following study of a list of related words (“dream,” “bed,” “night,” et cetera), suggesting that the representation of the lure was activated and erroneously selected during retrieval (Deese, 1959; Roediger and McDermott, 1995; Steffens and Mecklenbräuker, 2007). In these studies, false alarming is largely driven by semantic similarity of items, and generally occurs immediately following the study. In the current experiment, participants read sentences that were not semantically similar, and were tested after reading several items; thus, a finding of increased false alarming to lures would be a powerful demonstration of prediction’s lasting effects on recognition memory.

In addition to behavioral effects, we were also interested in the processes involved in false recognition, as revealed by electrophysiological responses; however, previous results of when and how false recognition manifests in the ERP have been mixed (Curran et al., 2001; Wolk et al., 2006; Geng et al., 2007; Beato et al., 2012; Chen et al., 2012). A recent ERP study showed that words that were previously expected, but not presented, elicited a “pseudo-repetition” effect (Rommers and Federmeier, 2018b); namely, these items showed ERP effects similar to repeated words, suggesting they were not fully suppressed. We hypothesized that, if similar processes also influence end-state recognition responses, these predicted but unobserved lures would show higher false alarms than new items. Furthermore, we used the N400 and LPC to help clarify the neurocognitive mechanisms involved in prediction-based false alarms vs. correct rejections of lures, focusing on whether these responses were associated with priming and/or recollection during the recognition test.

## Materials and Methods

### Participants

Thirty-three right-handed, native speakers of English with normal or corrected-to-normal vision from the University of Illinois, Urbana-Champaign participated in the experiment and were paid $10 an hour or received course credit for their participation. No participant had a history of neuropsychological or psychiatric disorders. Procedures were approved by the IRB of the University of Illinois, and all participants signed consent forms prior to participation. Based on previous work using these same materials to examine ERPs during sentence comprehension (Federmeier et al., 2007), the *a priori* number of subjects was set to 32; mid-way through data collection, a participant’s recorded data was noisy, and thus an extra subject was run. Data analysis led to the removal of another subject’s data due to high trial loss, leading to a sample size of 31 participants in the final analyses.

### Materials

The stimuli were comprised of 192 English sentences, a subset of the sentences used in Federmeier et al. (2007). The cloze probabilities of the endings of the sentences were previously determined in a norming study in which the subjects filled in the final word of the sentence frame with the word they “would generally expect to find completing the sentence fragment.” In the current experiment, half of the stimuli (96 sentences) were strongly constraining, while the other half were weakly constraining. A sentence was considered strongly constraining if the cloze probability of the most commonly completed word was 0.68 or higher, and was considered weakly constraining if the cloze probability was 0.42 or lower. Additionally, half of the strongly constraining sentences (48 sentences) ended with the expected word, while the other half ended with an unexpected word; this was also true of the weakly constraining sentences. Unexpected words all had a cloze probability close to 0 (max = 0.088). Thus, participants read 48 strongly constraining sentences with expected endings (SCE; mean cloze = 0.83), 48 with unexpected endings (SCU), 48 weakly constraining sentences with expected endings (WCE; mean cloze = 0.28), and 48 with unexpected endings (WCU). These stimuli were evenly split into eight blocks (six of each condition in each block). Table 1 provides the lexical properties (word frequency, concreteness, imageability, familiarity, word length) of the sentence ending words. Target words averaged 5–6 letters in length and were fairly concrete, imageable, and familiar; unexpected endings tended to be of lower frequency on average than expected endings but were similar across constraint.

**Table 1:** Lexical properties of sentence ending words.

Condition	Frequency	Concreteness	Imageability	Familiarity	Word length
SCE	4.16	506.10	527.31	574.00	4.98
SCU	3.06	502.45	520.82	554.67	6.29
WCE	3.96	501.10	518.38	575.94	5.52
WCU	3.22	485.83	520.19	554.027	5.85

*Values represent means across items. Frequency values are log transformed and obtained from Kučera and Francis (1967). Concreteness, imageability, and familiarity values obtained from the MRC psycholinguistic database*.

After each block of sentence reading, participants took a memory test. For the recognition memory test, participants were presented with single words, the majority of which were words that had ended the previously read sentences. “Matches” were words that had previously been seen as sentence endings (either expected or unexpected). “Lures” were words that might have been expected (they were the most likely completion of a sentence from the prior block) but were never actually presented (because the sentence had ended with an unexpected word instead). As an example of a “Lure” item, during the encoding phase a participant might read the sentence “I added my name to the basket,” where basket is an unexpected ending, and in the test phase read the word “list,” the expected ending of the sentence. “New” words had never been presented in the block. The test also contained some sentence-medial words, to ensure that participants would be motivated to pay attention to and encode the entire sentence. Half of the test items that were previously sentence ending words were from strongly constraining sentences while the other half were from weakly constraining sentences. Table 2 provides examples of the different types of test items. There was an equal number of items presented in each of the conditions during each test block (totaling 24 in each condition, along with 48 new items, over the course of the experiment), as well as an equal number of “old” and “new” items each test, so as not to bias responses. As with the sentence endings, lexical properties of the test items (see Table 3 for details) were similar across conditions, with some variation in frequency; we aimed to test the impact of the frequency variability in our statistical models.

**Table 2:** Examples of experimental materials.

SC	Tim threw a rock and broke the	E	window	(Match)	window
		U	camera	(Match)	camera
				(Lure)	window
WC	His ring fell into a hole in the	E	sink	(Match)	sink
		U	couch	(Match)	couch
				(Lure)	sink

*SC, strong constraint; WC, weak constraint; E, expected; U, unexpected. Match and Lure refer to the items that appear during the memory test*.

**Table 3:** Lexical properties of test words.

Condition	Frequency	Concreteness	Imageability	Familiarity	Word length
SCE Match	4.21	526.58	539.95	573.63	5.04
SCU Match	2.94	477.00	503.84	546.33	6.21
SC Lure	4.13	507.45	527.86	571.77	5.29
WCE Match	4.04	503.87	525.00	582.44	5.38
WCU Match	2.99	476.22	509.67	552.94	6.08
WC Lure	3.43	509.81	527.18	580.94	5.29
New	3.70	497.77	525.31	560.15	5.77

*Values represent means across items. Frequency values are log transformed and obtained from Kučera and Francis (1967). Concreteness, imageability, and familiarity values obtained from the MRC psycholinguistic database*.

The memory test constrained the stimuli used and the order of presentation, in that each test item had to be unique, as well as not repeat. For example, participants might read the sentence “he played with the dog,” see the word “dog” during the memory test, and later read the sentence “the dog ate the food.” To avoid participants reading both sentences before being tested, the stimulus list was pseudo-randomized, such that any sentence containing a critical test item in the middle of it was presented only after the item had already been tested. All participants read the same list of stimuli; although the order of presentation of each stimulus within blocks was randomized, the order of presentation of the blocks was not.

### Procedure

Participants were seated in an electrically shielded EEG recording booth approximately 100 cm from a CRT computer monitor. Prior to starting the experiment, we verified that all participants could easily read the presented information from this distance. Additionally, participants were given an explanation of the experimental procedure, as well as a short practice session to familiarize them with the task. Words that appeared in the practice sentences and test items did not appear as critical test words in the actual experiment.

The experiment was divided into eight study-test blocks, in which participants first studied a set of sentences, and then were tested on their memory for critical words. During the encoding phase of each study-test block, participants were instructed to read the sentences silently and to try to remember what they read, as their memory would be tested. Sentences were presented word by word on the screen, with each word appearing in the center of the screen for 200 ms, followed by a 300 ms interstimulus interval. After the last word of the sentence was presented, a blank screen was presented for 500 ms, followed by a fixation cross for 1,000 ms. Participants were instructed to try not to blink when they were reading the sentence, and to blink and rest their eyes once the fixation cross appeared. Following the encoding phase, participants were given math problems to complete for 30 s. The math problems were simply given as a distractor between the study and test phases—thus, performance on the math section was not analyzed.

After the math section, participants started the test phase of the block. Each trial began with a fixation cross in the center of the screen for 1,000 ms, which was then replaced by a test word. After 1,000 ms, a confidence scale appeared underneath the test word, at which point participants could make their response. Upon making a response, the trial would end and the next trial would begin. The confidence scale consisted of four points—“Sure New,” “Maybe New,” “Maybe Old,” and “Sure Old.” Participants were instructed to respond with “Old” if they thought the test word was a word they had seen during the encoding phase and otherwise to respond “New.” Additionally, they were told to try to use the whole scale of confidence and to use the “Maybe” option if they felt like they were guessing or unsure. Finally, participants were instructed to try not to blink during the initial presentation of the word, but told that once the confidence scale appeared and they could make their response, as well as during the fixation cross, they could blink. The test phase was self-paced, in that participants could take as long as they needed to respond.

### EEG Recording and Processing

EEG data were recorded from 26 Ag/AgCl electrodes embedded into a flexible elastic cap and distributed over the scalp in an equidistant arrangement; see icon in Figure 2. Five additional electrodes were attached, including one on each mastoid bone behind the ear, one adjacent to the outer canthus of each eye, used for monitoring of the horizontal electro-oculogram (EOG), and one below the lower eyelid of the left eye, used for monitoring of blinks. Electrode impedances were kept below 5 kΩ. Signals were amplified by a BrainVision amplifier with a 16-bit A/D converter, an input impedance of 10 MΩ, a bandpass filter of 0.016–100 Hz, and a sampling rate of 1 kHz. The left mastoid electrode was used as a reference for on-line recording; offline, the average of the left and right mastoid electrodes was used as a reference.

**Figure 1:**
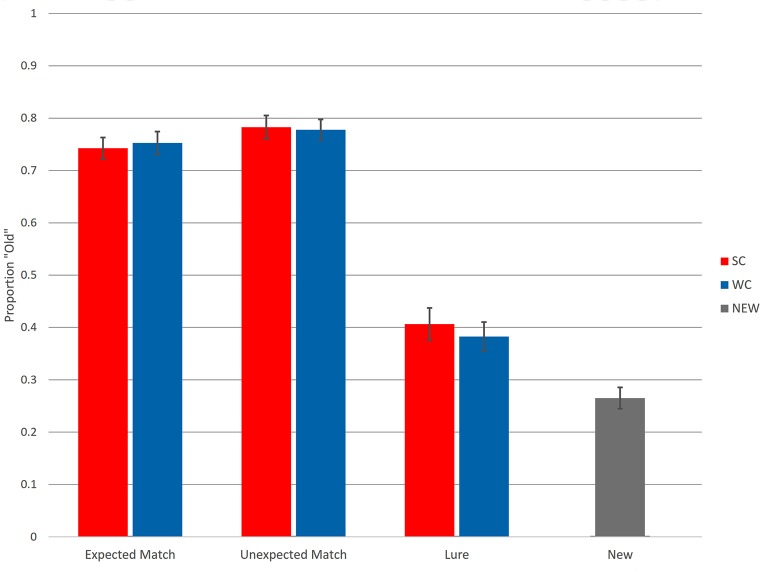
Recognition memory accuracy. Proportion “Old” responses are plotted on the Y axis. SC, strong constraint; W, weak constraint. Error bars reflect standard error around the mean.

**Figure 2:**
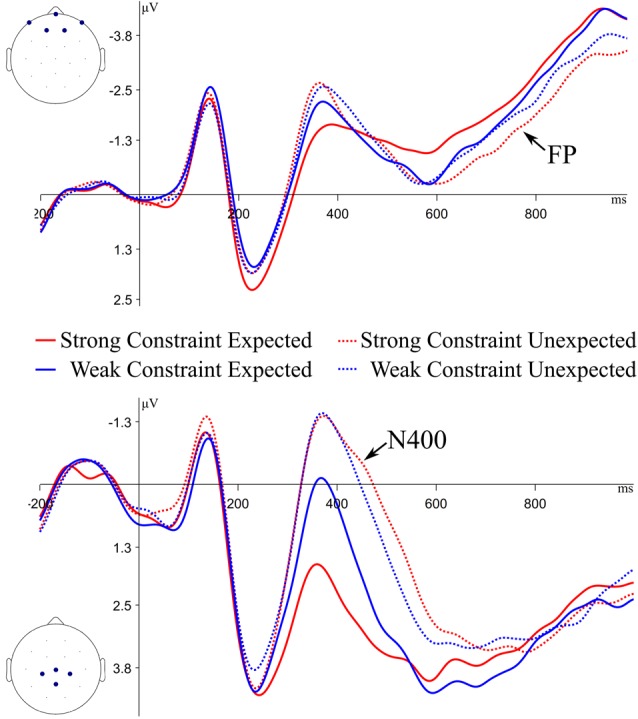
Grand average event-related potential (ERP) waveforms for expected and unexpected endings to strongly and weakly constraining sentences at the frontal Cluster (top of figure) and central Cluster (bottom of figure) of channels. Negative is plotted up. FP, frontal positivity.

Following data collection, each raw EEG time series was passed through a 0.1–30 Hz Butterworth filter with a 12 dB/oct roll-off. The signal was segmented into epochs from −200 to 1,000 ms relative to the onset of each sentence ending word during encoding and each test item during the test phase. Following subtraction of the 200 ms prestimulus baseline, and artifact correction (described below), epochs within each bin were averaged together to create an ERP for each subject and bin. Prior to calculating statistics, individual subject ERPs were passed through an additional 20 Hz lowpass filter.

To correct for ocular artifacts, a bipolar VEOG channel was created by subtracting data in the lower eye channel from the most frontocentral channel (MiPf), and that channel was then scanned with a sliding window step function to detect blinks. For subjects who had a large number of blinks, the data were run through AMICA (Palmer et al., 2011), an ICA decomposition algorithm that generalizes Infomax and multiple mixtures approaches adaptively. Following decomposition, the correlation between the timecourse of each component and the VEOG channel was calculated in order to find the component(s) containing blinks. Components with a high correlation were removed from trials marked as containing blinks. The remaining components were then recombined to reconstruct the EEG data, which were then scanned with an additional sliding window amplitude threshold (300 ms sliding time window, 50 ms step size, 90 μV threshold), and finally manually checked by the experimenter for any additional artifacts. In total, an average of 8% of trials were removed, with a range of 2% to 11% across participants. Artifacts were spread fairly evenly across conditions, resulting in an average of 22 trials in each condition of the memory test.

**Figure 3:**
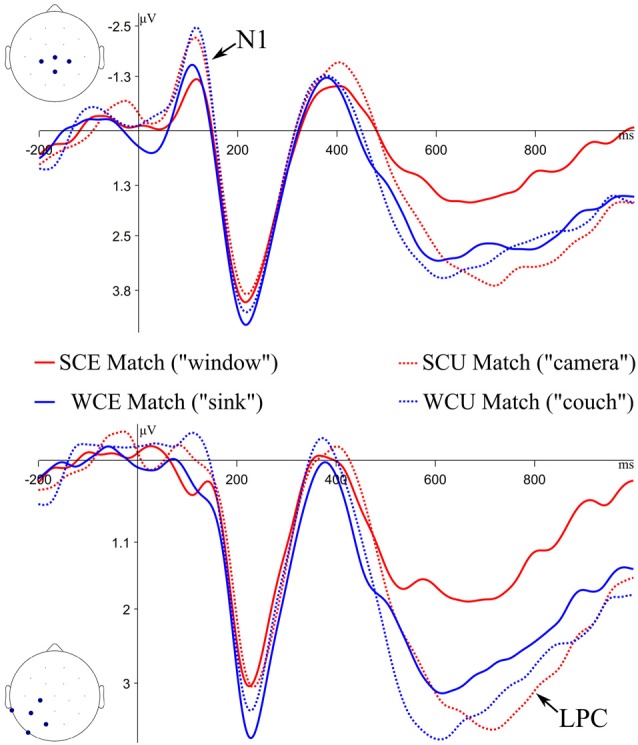
Grand average ERP waveforms to Match items during the memory test. ERPs plotted at the Central Cluster (top of figure) and the Left Parietal Cluster (bottom of figure). SC, strong constraint (“Tim threw a rock and broke the…”); WC, weak constraint (“His ring fell into a hole in the…”); E, expected; U, unexpected. In quotations are example stimuli, based on examples from Table 1.

For the ERP analyses, statistical analyses were performed on channel clusters as opposed to single channels to improve the signal to noise ratio. Component-based analyses were done using the signal-averaged across channel clusters and time windows based on prior work: N400 at a central cluster (shown in Figure 2; Federmeier et al., 2007), 300–500 ms; frontal positivity at a frontal cluster, 700–1,000 ms (shown in Figure 2; DeLong et al., 2014); LPC at a left parietal cluster (shown in Figure 3; Woroch and Gonsalves, 2010; Addante et al., 2012), 500–800 ms. For other effects, as described in the results, cluster-based permutations with restricted time windows were used in order to explore the data while retaining statistical power and maintaining Type I error rate (Fields and Kuperberg, 2018). Plotted ERPs were filtered with a 10 Hz lowpass filter for clarity of visualization.

## Results

### Behavior

Proportion “Old” responses is plotted in Figure 1. For Matches, “Old” was a correct response, whereas for New items and Lures, “Old” was an incorrect response. Analyses revealed no differences in confidence across experimental conditions and generally low trial numbers for “Maybe” responses; thus, “Maybe” responses were combined with “Sure” responses for behavioral and ERP analyses. Overall, participants successfully discriminated Matches from New items. Collapsing across Match conditions and comparing to New items, the average *d*′ was 1.41, with a range of 0.72–2.76. Recognition accuracy between Expected and Unexpected Matches appeared similar, whereas participants false alarmed more to Lures compared to New items.

To assess the pattern statistically, behavioral responses (Old or New) on each trial were submitted to a mixed-effects logistic regression model fit by maximum likelihood using the lme4 package in R (Jaeger, 2008). Random factors included intercepts for items and slopes and intercepts for participants for each fixed effect. Correlations between random factors were not calculated to ease convergence of the models. Wald’s *z*-scores were computed for each coefficient to test for significance.

The first model compared responses to Lures with responses to New items by modeling responses to those items with Condition (Lures, New) as a fixed factor. Recognition accuracy differed between Lures and New items (*β* = 0.75, *z* = 3.18, *p* < 0.01), but accuracy did not differ between Strong Constraint Lures and Weak Constraint Lures (*β* = 0.11, *z* = 0.42, *p* = 0.68). Thus, participants showed greater false alarms to Lures compared to New items.

Although we attempted to control the lexical properties of stimuli, it could be the case that a subset of the Lures were more frequent than other Lures or New items, and this could have contributed to the false alarm effect. To assess this, a second model was fit with Condition and log-transformed Word Frequency as fixed effects. Frequency had a significant effect on responses (*β* = 0.30, *z* = 4.72, *p* < 0.01), with higher Frequency leading to a greater number of “Old” responses, but recognition accuracy still differed between Lures and New items (*β* = 0.64, *z* = 2.91, *p* < 0.01). Thus, word frequency did not completely explain the false alarm effect that we observed.

The next model assessed responses for Matches by modeling responses with Constraint, Expectedness, and the interaction (Constraint * Expectedness) as fixed factors. None of the coefficients, Constraint (*β* < 0.01, *z* = 0.06, *p* = 0.95), Expectedness (*β* = 0.21, *z* = 1.26, *p* = 0.21), or the interaction (*β* = 0.13, *z* = 0.27, *p* = 0.79), returned significant *z*-scores. Including word frequency in the model (C*E*F) did not change previous results, although word frequency seemed to have a tendency to reduce “Old” responses (*β* = 0.10, *z* = −1.94, *p* = 0.05). Thus, behavioral accuracy for Match items did not differ based on constraint or expectedness.

### Sentence Final Word ERPs

ERPs to sentence final words were analyzed to determine if prior effects seen with these materials (e.g., Federmeier et al., 2007) were replicated. Grand average ERPs at the sentence final word at the frontal and central cluster are plotted in Figure 2. To assess effects statistically, linear mixed-effects models were used (Baayen et al., 2008), using the lme4 and lmerTest packages in R. Random factors included intercepts for items and slopes and intercepts for participants. As with the behavioral analyses, correlations between random factors were not calculated to ease convergence of the models. The reported *t*-tests used the Satterthwaite approximations to calculate degrees of freedom (Satterthwaite, 1946).

N400 amplitudes were compared between weakly constrained expected (WCE) endings and strongly constrained expected (SCE) endings, as well as between WCE and unexpected (U) endings (collapsed across constraint, as this has repeatedly been shown not to affect N400 responses). There were significant differences in N400 amplitude between WCE and SCE endings (*β* = 1.32, *t* = 2.87, *p* < 0.01), as well as between WCE and U endings (*β* = 1.70, *t* = 4.42, *p* < 0.01). Thus, the graded N400 effect was replicated in this experiment.

ERPs to sentence final words were also analyzed to determine if the frontal positivity to Strong Constraint Unexpected endings was replicated. The frontal positivity has been operationalized as a difference between Strong Constraint Unexpected (SCU) and Weak Constraint Unexpected (WCU) endings (Federmeier et al., 2007), or a difference between expected (E) endings and SCU endings (DeLong et al., 2014), so both of these differences were tested. There were no significant differences in frontal positivity amplitudes between the SCU and WCU conditions (*β* = 0.41, *t* = 0.95, *p* = 0.35); however, SCU endings elicited larger positivities than E endings (*β* = 0.84, *t* = 2.01, *p* = 0.05). A follow-up comparison of WCU and E conditions showed no significant differences (*β* = 0.42, *t* = −1.04, *p* = 0.31). Thus, the frontal positivity from SCU endings was more positive than other conditions, replicating prior work, but did not differ significantly from the WCU condition.

### Recognition Memory ERPs: Matches

ERPs to correctly recognized test items were analyzed to assess recognition memory processes. The grand average ERPs at the central cluster to expected and unexpected Matches from strongly and weakly constraining sentences are plotted in Figure 3. ERPs are time-locked to the onset of the test item, and only correct responses are included.

LPC mean amplitudes from 500 to 800 ms at the Left Parietal cluster were submitted to a linear mixed effect model with fixed effects of Expectancy (E vs. U) and Constraint (SC vs. WC). The fixed effect of Expectancy was significant (*β* = 1.14, *t* = 2.53, *p* = 0.01), whereas Constraint (*β* = 0.51, *t* = −0.97, *p* = 0.34) and the interaction (*β* = 0.80, *t* = 0.94, *p* = 0.35) were not. A follow-up comparison of LPCs from SCE Matches and WCE Matches trended toward significance (*β* = 0.94, *t* = −1.93, *p* = 0.06). Unexpected Matches generated more positive LPC amplitudes compared to Expected Matches, and SCE Matches generated the smallest LPC amplitudes.

Visual inspection suggested an additional effect on the N1, a component that is part of the visual evoked potential and is sensitive to attention (Mangun and Hillyard, 1991). To assess this effect, we performed a *post hoc* exploratory analysis, using a cluster-based permutation test with a restricted time window based on previous literature to increase statistical power (Maris and Oostenveld, 2007; Groppe et al., 2011; Fields and Kuperberg, 2018). In this test, *t*-tests were calculated at each time-point and channel, and significant *t*-values that were adjacent in space and time were clustered together. Clusters were characterized by taking the sum of *t*-values within the adjacent points. These observed clusters were compared to a permutation distribution, generated by shuffling the condition labels of the data, finding clusters, and summing the *t*-values of the clusters 2,500 times. Distributions of the most extreme cluster sums were created for comparison to the observed cluster sums. Reported *p-values* represent the percentile ranking of the observed clusters compared to the permutation distribution. Here, *t*-tests tested differences between Expected Matches and Unexpected Matches at each channel and time-point within the 50–175 ms window, and a family-wise alpha of 0.05 was used.

**Figure 4:**
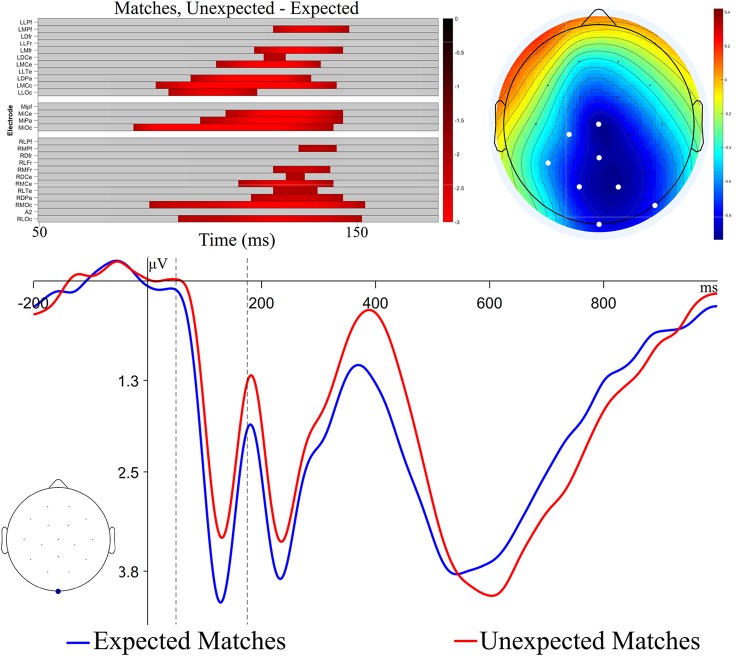
Permutation test results and ERP plots for analyses of N1 recognition memory effect. The raster plot show channels and time-points which make up the significant cluster found in the permutation tests. Colors represent the *t*-value at the time-point. The ERP topography plot shows the mean amplitude in the time window of the significant cluster, with significant channels highlighted in white. The ERP plot shows the Expected and Unexpected Match ERPs at the channel with the largest *t*-value within the cluster (MiOc). The black dashed lines indicate the time range of the permutation test.

The results of this analysis are displayed in Figure 4. A significant difference between Expected and Unexpected Matches was found (cluster-wise *p* < 0.05). This difference had a temporal extent from 81 to 153 ms and a central-posterior topography, similar to previously reported posterior visual N1 effects, though somewhat earlier in time (Di Russo et al., 2001; Hopf et al., 2002). Thus, Unexpected Matches elicited more negative N1 potentials compared to Expected Matches^1^. To test for the possibility of pre-stimulus activity leading to the appearance of an N1 effect, an addition permutation test was run on the same contrast in the 0–80 ms time window. No significant clusters were found (*p* = 0.29).

### Recognition Memory ERPs: Lures

Of particular interest for the analysis of ERPs to Lures was if ERPs differed between false alarms and correct rejections, and whether this ERP difference was affected by constraint. However, few studies have investigated ERP differences to false alarms and correct rejections, particularly for previously predicted information. Thus, while we were interested in early vs. late differences, there were not* a priori* predictions about particular ERP components to target in the post-N400 time window. We thus used time-constrained permutation tests, as described for the N1 analyses (Fields and Kuperberg, 2018). ERPs to SC and WC Lures were separated into Correct and Incorrect bins based on the response given (pooled across “Maybe” and “Sure”), and the difference between these ERPs was calculated. These difference waves were submitted to cluster-based permutation tests to test time-points for significant differences from 0, using a family-wise alpha value of 0.05. Separate permutation tests were run for Strong Constraint and Weak Constraint lures, and to increase statistical power and focus on times of interest, separate permutation tests were run for time windows of 300–500 ms (N400) and 500–1,000 ms.

**Figure 5:**
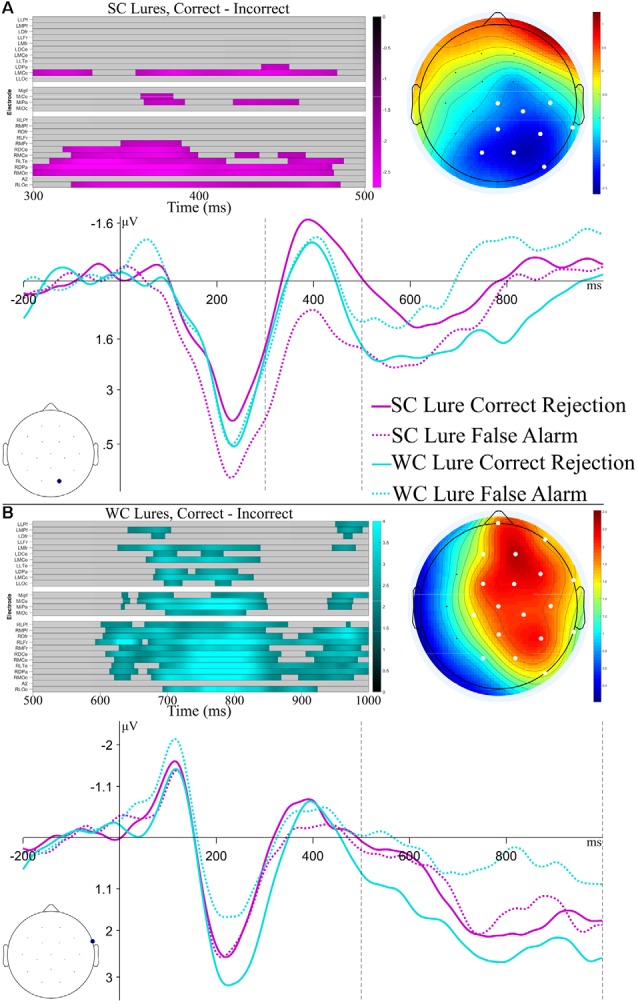
Results and ERP plots for analyses of Lures. The top half **(A)** focuses on Strong Constraint Lures, with a time window of 300–500 ms, whereas the bottom half **(B)** focuses on Weak Constraint Lures, with a time window of 500–1,000 ms. The raster plots show channels and time-points which make up the significant cluster found in the permutation tests. Colors represent the *t*-value at the time-point. The ERP topography plots show the mean amplitude in the time window of the significant cluster, with significant channels highlighted in white. The ERP plots show the SC and WC Lures at the maximal channel within the observed cluster. The black dashed lines indicate the time range of the permutation tests.

Results of the permutation tests and ERPs are plotted in Figure 5. For the Strong Constraint Lure comparison, a significant difference (cluster-wise *p* = 0.04) between false alarms and correct rejections was found in the 300–500 ms time window, while no significant differences were found in the late window. This difference began from the onset of the analysis window and continued to 488 ms, with a central-posterior topography. For the Weak Constraint Lure comparison, a significant difference (cluster-wise *p* < 0.01) between false alarms and correct rejections was found in the late time window, while no significant differences were found in the earlier window. This cluster showed a broad right-lateralized topography, with a right frontal maxima, and a temporal extent of 594–1,000 ms. These results suggest that mechanisms with different timecourses led to false alarming based on the constraint of the item^2^.

**Figure 6:**
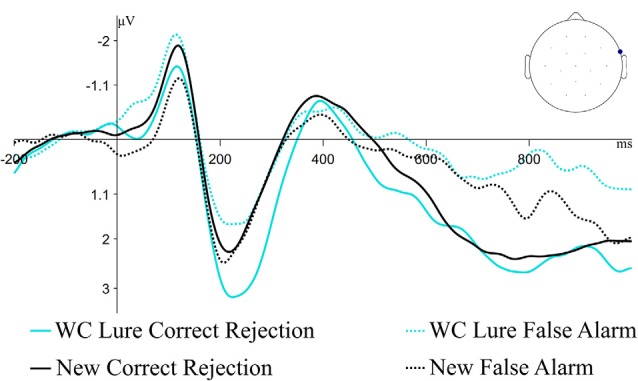
Grand average ERP waveforms for correct rejections and false alarms for Weak Constraint Lures and New items at the previously described maximal channel from the WC Lure cluster analysis. The pattern of responses for New items appears similar to the WC Lure items.

The behavioral effect of interest was the comparison of false alarm rates of Lure items compared to false alarm rates of New items; therefore, we were also interested in how the electrophysiological differences associated with false alarming to Lures compared to those associated with false alarming to New items. Figure 6 plots correct rejection and false alarm ERPs for Weak Constraint Lures as well as New items; although the ERPs at the same channel as before are plotted, the ERP patterns between these conditions were fairly similar across other channels as well. Permutation tests testing for differences between correct rejections and false alarm ERPs to New items in both the 300–500 ms and 500–1,000 ms windows were not significant (early, *p* = 0.09; late, *p* = 0.11), but numerically, false alarming to Weak Constraint Lures seemed to have engaged similar neurocognitive processes as false alarming to New items.

## Discussion

In this study, participants read strong and weak constraint sentences that ended with either an expected or unexpected-but-plausible word and then were tested on their memory for sentence ending words, new words, and predictable endings that had never been seen (lures). ERP responses during sentence reading replicated previously shown effects. We observed a graded N400 pattern (Federmeier et al., 2007), such that N400s were smallest to expected items in strong constraint sentences, intermediate to expected items in weak constraint sentences, and largest to unexpected items. We also found a post-N400 frontal positivity, larger for unexpected than expected words and numerically largest for unexpected words in strongly constraining sentences (where predictions can be correspondingly stronger). Different from the pattern in Federmeier et al. (2007), we did not observe a significant difference between unexpected items in strongly and weakly constraining contexts, seemingly because there was also some level of frontal positivity for the unexpected items in the weakly constraining sentences. It is possible that the memory task induced different reading strategies than the passive comprehension task in Federmeier et al. (2007). For example, Brothers et al. (2017) reported a larger frontal positivity to unexpected words when participants were instructed to predict upcoming information compared to when they simply read for comprehension. Anticipating an imminent memory test may have encouraged participants to read more attentively and devote more resources to prediction.

The central question for this study concerned participants’ later memory for sentence-ending words they had predicted and/or read. Behaviorally, hit rates were numerically higher for unexpected than for expected matches, though no reliable effect was found. A similar pattern had previously been seen for word recognition at the end of the experiment using these stimuli; higher hit rates were also found for expected words that had completed weakly vs. strongly constraining sentences (Federmeier et al., 2007; see also Corley et al., 2007). The ERPs during the memory test in the present study, however, revealed that LPC responses elicited by unexpected Matches were more positive than those to expected Matches, suggesting greater recollection for unexpected words. Additionally, LPC amplitudes differed between strongly and weakly constrained expected matches, with more positive LPCs for weak constraint matches. This LPC pattern mirrors the behavioral memory performance pattern observed in Federmeier et al. (2007). This pattern may arise because prediction trades off with depth of encoding, such that participants process—and hence encode—predicted words less. In other words, the information needed to verify that an expectation is met may require less attention and less stimulus-driven processing than that needed to encode a stimulus that readers could not predict. A recent ERP repetition study supports this account (Rommers and Federmeier, 2018a). Words that had first been encountered as expected sentence endings of strongly constraining sentences showed reduced ERP repetition effects (when seen again in a weakly constraining sentence) compared to those that had first been seen in weakly constraining sentences. Thus, predictability may have downstream costs: when information is pre-activated, comprehension may take place in a top-down “verification mode” (Van Berkum, 2010), in which readers need only confirm that the stimulus matches with the expectation. This process achieves speedier processing in the moment by sacrificing thorough processing of the bottom-up input, ultimately leading to impoverished representations. Future studies investigating memory for predicted information could assess this further by examining ERPs for misses or incorrect responses, as trial numbers were too low to assess misses here.

Surprisingly, unexpected matches also elicited larger (more negative) N1 amplitudes than did expected matches. N1 amplitude modulations are not routinely reported in electrophysiological studies of recognition memory. Although unexpected sentence endings may have received greater depth of processing during encoding, ERP studies examining retrieval of words that were deeply or shallowly encoded have not reported modulations of the N1 (Rugg et al., 1998, 2000; Allan et al., 2000). However, N1 modulations have been observed in the context of visual attention and categorization. The N1 is sensitive to the allocation of attentional resources (Mangun and Hillyard, 1991; Hillyard and Anllo-Vento, 1998) and may reflect an early, attention-dependent visual discrimination process that is sensitive to category membership (Vogel and Luck, 2000; Hopf et al., 2002). In one study (Curran et al., 2002), participants were trained in separating abstract blob images into two separate categories (similar or dissimilar to a prototype) and were later given a recognition memory test on the images. The N1 during the recognition test was sensitive to category membership, but not to old/new differences, similar to the current reported results. Differences in predictability during sentence reading may have led to separable categories during recognition testing; however, given the *post hoc* nature of the analysis of the N1 in the current study, it will be important to replicate the effect in future work, as well as to confirm that the results cannot be explained by other factors (such as lexical variables).

A critical manipulation in the current study was the inclusion of lures—items that were likely to have been predicted during sentence reading but that were never actually presented (because an unexpected word appeared instead). Behaviorally, individuals were significantly more likely to false alarm to Lures than to New items that had not been studied, suggesting increased accessibility or fluency for these items. This pattern is consistent with claims that words are predicted and pre-activated as a sentence unfolds (Federmeier, 2007; Kutas et al., 2011) and further reveals that such predictive pre-activation can have long-lasting effects. Here, several sentences were presented in each block, and each block was followed by interfering math problems, and yet participants still showed increased false alarming to these lures. This finding mirrors previously reported effects from studies on false memory using the DRM paradigm, in which subjects falsely recall—and are more likely to falsely recognize (Gallo, 2010)—critical lures that are semantically similar to studied items. However, a number of differences between the paradigms make the current findings particularly striking. First, in DRM experiments, the lure items are usually closely related to an entire list of words. Here, instead, each predicted sentence ending used as a Lure test item was related to only one sentence in a block, and the sentences were not semantically related to each other. Moreover, different from the DRM paradigm, in the present study predictions were explicitly disconfirmed, *via* the presentation of an unexpected word (which was always semantically unrelated to the predicted ending). Thus, these findings suggest that expected representations are not fully suppressed when a prediction is disconfirmed and that false memories can arise for such disconfirmed information. This presents another cost of prediction during language comprehension: individuals may falsely remember reading or hearing words that were not actually experienced, simply because they were predicted in the moment, and those predictions linger.

An alternative explanation of the luring effect is that participants could have tried to use the word presented during the test as a cue to perform a retrospective search through memory for a sentence that might have included it. By this account, when a Lure was presented, subjects were able to retrieve a likely sentence frame for that word, and thus more false alarming occurred. Similar to Neely and Keefe’s (1989) hybrid prospective-retrospective processing theory, this retroactive search could be performed regardless of any pre-activation of the test item. However, in the case of the Lures in the present study, the associated sentence was completed by an unexpected word. For a retroactive search strategy to work, the unexpected word that originally completed the sentence and its effect on the sentence-level meaning that was extracted would need to be ignored, thus rendering the Lures as ineffective search cues.

Behaviorally, participants did not show a greater rate of false alarms to lures from strongly constraining sentences compared to lures from weakly constraining sentences. However, electrophysiological analyses revealed that different underlying patterns of brain activity were associated with false alarming across constraint. False alarming to strong constraint lures correlated with an earlier, N400-like effect, whereas false alarming to weak constraint lures was associated with a later, right-lateralized effect that was fairly broadly distributed. The N400-like pattern to the lures from the strong constraint sentences is consistent with the idea that false alarms to these items were driven by an increase in conceptual fluency or familiarity (Voss and Federmeier, 2011; Wang et al., 2015). A plausible account of this effect is that when words or concepts are strongly predicted, they linger, such that when the word is encountered again, it is processed more fluently or is more familiar, which behaviorally is associated with a tendency to mark these words as “old” and electrophysiologically is associated with a reduced N400 response. The later right-lateralized effect observed following false alarms to weak constraint lures may be comparable to the right frontal old/new effect in the recognition memory literature, which is thought to index decision making, evaluation, and post-retrieval monitoring processes (Hayama et al., 2008; Cruse and Wilding, 2009; Hayama and Rugg, 2009) and has been related to lure discrimination (Morcom, 2015). Thus, despite a lack of behavioral differences in false alarming based on constraint, it appears different processes may have led to false alarms depending on the prior constraint of the item: a more rapid semantic matching based process for strong constraint lures and a slower, more top-down decision process for weak constraint lures. Future studies could use experimental manipulations to dissociate these effects; for instance, employing speeded recognition decisions would likely increase false alarm rates for weak constraint lures, but might not affect strong constraint lures.

Overall, these results demonstrate that prediction during language comprehension has important downstream effects on recognition memory. Participants were more likely to false alarm to predictable, but never observed words compared to unexpected and unstudied words, suggesting unobserved predictions are not fully suppressed and remain accessible in memory. Individuals also had enhanced memory for unexpected information, as evidenced by larger LPC amplitudes during recognition testing, along with a larger N1 response. Finally, ERPs revealed sentential constraint-based differences in the neurocognitive mechanisms involved in false alarming to lures, with earlier semantic matching processes contributing to false alarms to strongly predicted information, but later decision-making processes contributing to false alarms to weakly predicted information. Ultimately, prediction during language comprehension does have costs: namely, predicting upcoming words in sentences can produce more rapid processing in the moment, but can lead to impoverished memory of predictable information and false remembering of unobserved predictions.

## Data Availability

The datasets generated for this study are available on request to the corresponding author.

## Ethics Statement

### Human Subject Research

The studies involving human participants were reviewed and approved by University of Illinois Institutional Review Board. The patients/participants provided their written informed consent to participate in this study.

## Author Contributions

RH, JR, CJ, and KF contributed to the conception, design of the study and wrote the manuscript. RH and JR collected data. CJ created code for generating stimulus lists with non-repeating stimuli. RH performed the statistical analysis. All authors contributed to manuscript revision, read and approved the submitted version.

## Conflict of Interest Statement

The authors declare that the research was conducted in the absence of any commercial or financial relationships that could be construed as a potential conflict of interest.

## Footnotes

^1^A mixed effect analysis was also run on single trial N1 amplitudes derived from significant cluster timepoints and channels, with fixed effects of expectancy and word frequency, to control for lexical confounds. The effect of expectancy was significant (*β* = 0.21, *t* = 3.41, *p* < 0.01), while frequency was not (*β* = 0.03, *t* = 1.53, *p* = 0.13). However, since estimates were derived based on cluster analyses, this mixed effect analysis could be considered double-dipping, and further replication of this effect will be necessary.

^2^Mixed effect analyses were also run on single trial SC and WC lure amplitudes derived from significant clusters, with fixed effects of correct/incorrect and word frequency. For both analyses, the fixed effect of correctness was significant (SC: *β* = 0.36, *t* = 2.64, *p* = 0.01; WC: *β* = 0.87, *t* = 3.61, *p* < 0.01), while word frequency was not significant (SC: *β* = 0.09, *t* = 1.45, *p* = 0.16; WC: *β* = 0.05, *t* = 0.72, *p* = 0.48). As with the N1 effect, these results could be considered double dipping and replication will be necessary.
